# Marine Environmental Knowledge and Attitudes among University Students in Hong Kong: An Application of the Ocean Literacy Framework

**DOI:** 10.3390/ijerph20064785

**Published:** 2023-03-08

**Authors:** Debbrota Mallick, Eric Po Keung Tsang, John Chi-Kin Lee, Chi Chiu Cheang

**Affiliations:** 1 Department of Science and Environmental Studies, The Education University of Hong Kong, Hong Kong SAR, China; 2 Stokes School of Marine and Environmental Sciences, University of South Alabama, Mobile, AL 36688, USA; 3 Dauphin Island Sea Lab, Dauphin Island, AL 36528, USA; 4 Department of Curriculum and Instruction, The Education University of Hong Kong, Hong Kong SAR, China

**Keywords:** marine environment, knowledge, attitudes, demographic variables, university student, curricular involvement

## Abstract

In this study, we assessed the general marine environmental knowledge and attitudes of university students from eight public universities in Hong Kong. The Ocean Literacy Framework and revised New Ecological Paradigm (NEP) were used as tools for questionnaire development. Data were collected via in-person and online surveys. An in-person survey was conducted at the university canteen from 16 May to 24 May 2017, and an online survey was conducted via email from 1 May to 31 May 2017. A structured questionnaire was provided to interested students from different levels of study and majors. Data obtained from these surveys were summarized based on participants’ correct answers in the general knowledge section and five-point Likert scaling for attitude statements. Results show that Hong Kong university students possess moderate marine environmental knowledge and pro-environmental attitudes. Knowledge scores significantly correlate with demographic variables, such as major of study, gender, institution, and parents’ education. Students’ pro-environmental attitudes are associated with different factors, including participation in various marine recreational activities, taking marine-related courses, and attachment to conservative marine initiatives. The study results have implications regarding the advancement of marine environmental knowledge and the pro-environmental attitudes of university students, such as mapping a well-structured pathway for disseminating marine environmental knowledge, curricular involvement, and the development of an integrated web resource.

## 1. Introduction

The ocean is a key feature of the planet, covering almost 71% of the Earth’s surface and providing essential ecosystem services to humanity [1,2]. Terrestrial life is predominantly influenced by the ocean, as it regulates climate, supports livelihood, provides food and non-food biotic raw materials, and facilitates recreation and other activities [3]. The ocean’s major climatic supports include controlling atmospheric heat, acting as a significant energy source, carbon sinking, and releasing oxygen [4].DeVries et al. [5] recently estimated that oceans can absorb 40% of anthropogenic carbon dioxide, indicating the oceans’ role in absorbing greenhouse gases. However, the marine ecosystem faces significant threats, such as ocean acidification [6], plastic pollution [7], overfishing, and harmful algal blooms [3], as a part of anthropogenic stresses on the oceanic system.

Several efforts have been made to facilitate marine environments in the past, and their improvement has continued in recent decades. The United Nations’ response in establishing sustainable development goal 14 was a promising effort to conserve the oceans [8]. This goal aims to conserve and ensure the sustainable use of marine resources. Unfortunately, recent performance scores show the worst rating for high-income countries [9], including some alarming trends for different countries concerning biodiversity conservation and climate change both underwater and on land. The United Nations General Assembly recently proclaimed a decade of ocean science (2021–2030) to strengthen the previous efforts regarding marine conservation. The primary concern of this declaration is raising awareness about the swift deterioration and overuse of marine resources [10]. However, the decade’s success solely depends on the potentiality of global capacity building and cooperation among countries. Therefore, a significant development in ocean education was emphasized at all levels among different stakeholders.

The successful dissemination of marine environmental education requires the assessment of existing knowledge among stakeholders. However, it has been debated how much marine environmental knowledge is required for citizens to be ocean-literate. The concept of ocean literacy provides an effective framework for understanding the ocean’s influence on human beings and vice versa [11]. An ocean-literate person can (i) “comprehend the important principles and fundamental concepts about the ocean”, (ii) “communicate through the ocean-related things in a meaningful way”, and (iii) “make informed and responsible decisions regarding marine resource utilization” [11]. Knowledge is the first potential stride in this regard and has been recognized as the center of the policy-making process by many scholars [12]. Remarkably, a person cannot be environmentally literate without knowledge of marine and aquatic systems. These are the “conceptual glue” that helps hold the Earth’s science system together [13]. In this study, we used an ocean literacy framework to assess university students’ general marine environmental knowledge.

The Ocean Literacy Framework comprises 45 fundamental concepts; this tool has been successfully used in many studies [14,15,16]. Ocean literacy is defined as “the understanding of the ocean’s influence on you and your influence on the ocean”. The seven principles of the ocean literacy framework [17] are:

i. The Earth has one big ocean with many features;
ii. The ocean and life in the ocean shape the features of the Earth;
iii. The ocean has a significant influence on weather and climate;
iv. The ocean made the Earth habitable;
v. The ocean supports a great diversity of life and the ecosystem;
vi. The ocean and humans are inextricably interconnected;
vii. The ocean is largely unexplored.

A validated assessment protocol should follow these seven fundamental principles of the framework to gauge a person’s ocean literacy [18]. Greely [16] successfully used these framework principles to measure ocean literacy by developing a set of 57 multiple-choice questions. Furthermore, Mogias et al. [19] used a modified version of Greely’s questionnaire with 66 multiple questions to assess the marine environmental knowledge of Greek pre-service teachers. Markos et al. [18], Guest et al. [20], Mogias et al. [21], Chang [22], Fauville et al. [23], Tsai and Chang [24], and others later successfully adjusted this ocean literacy framework either by developing a new assessment tool or by applying the existing framework to determine the ocean literacy of both students and the public. The use of ocean literacy in marine education has increased notably since 2013. Most studies using this framework were conducted in the USA [25]. However, ocean literacy has been successfully measured in other localities, such as the United Kingdom, Canada, Greece, Spain, and China. Data on the ocean literacy of primary (elementary) school students is scarce. A recent study showed a moderate level of knowledge among 1004 students from European countries [21]. Studies are relatively abundant at the high school level, although most have involved the development of assessment instruments, e.g., [22,23,24], and only a few have concentrated on studying the literacy level of participants.

Greely [16] used the ocean literacy framework to study the knowledge acquisition of 30 female students who joined an oceanographic camp. The results showed a notable improvement in students’ marine knowledge after the camp program. Plankis and Marrero [26] observed similar results in a program sponsored by the NOAA. About 464 K-12 students improved their knowledge of general marine environmental affairs. Canadian school students in grades 7–12 achieved poor results in answering the ocean literacy instrument despite their positive valuation of the marine environment. In contrast to primary and secondary schools, ocean literacy is poorly used in assessing university students’ ocean literacy. Mogias et al. [19] observed moderate ocean literacy among 421 Greek pre-service primary school teachers. Chen and Tsai [14] found positive attitudes and moderate level of marine knowledge among Taiwanese university students, Umuhire and Fang [27] observed a lack of knowledge to initiate a willingness to participate in marine-related actions. University students are the future decision makers in science and environmental operations. Improving ocean literacy at the tertiary level is therefore essential to ensure the application of sustainable approaches in marine affairs. Strengthening ocean literacy at the tertiary level demands a proper integration with the primary and secondary levels of study. Therefore, more studies on university students’ marine environmental knowledge using the Ocean Literacy Framework will help to identify the appropriate channels for disseminating marine environmental knowledge at the tertiary level via effective integration with the primary and secondary levels.

Sustainable use of marine resources solely depends on people’s understanding of human–sea interactions. Coastal cities and countries are the key places to deal with such issues in terms of ocean literacy. As an international business hub, Hong Kong has significant influences on various ocean-related sustainability issues from a global perspective, such as unsustainable fishing activities [28], overconsumption, and trading of declining global marine resources, including shark fin [29] and live reef fish [30]. In addition, the huge demand for seafood in Hong Kong and mainland China exerts pressure on global marine ecosystems [31]. Furthermore, seafood trading is prominent in southern China [32,33]. Therefore, residents’ ocean literacy (including school and university students), is vital in ensuring sustainable use of marine resources. To date, no published literature has gauged the ocean literacy of Hong Kong citizens and students. However, studies on pressing ecological issues such as shark conservation [34] have revealed several misconceptions and insufficient knowledge among primary students. On the other hand, participation in interpretative programs such as Chinese white dolphin watching and eco-garden-based programs was found to enhance participants’ ecological knowledge and environmentally responsible behavior [35,36,37]. Hence, different possible routes are needed to improve ocean literacy among students and citizens. To this end, understanding the current level of ocean literacy among these stakeholders is essential.

Given the adverse impacts and potential data gap, the current study aims to assess the ocean literacy of university students from Hong Kong. As a cosmopolitan city developed from a traditional fishing port, data on Hong Kong could contribute to filling this knowledge gap to enhance ocean literacy globally. Hence, it is important to assess the ocean literacy of university graduates, as their contribution can be crucial in strengthening ocean literacy among general citizens and identifying knowledge gaps in the academic system (primary school to university).

## 2. Methodology

### 2.1. Sampling Design

There are about 20 local higher-degree-awarding institutes in Hong Kong, 8 of which are UGC-funded public universities [38]. Data were collected from the eight public universities (Table 1). The total target population was 89,600 full-time and 3800 part-time students [38]. To determine the appropriate sample size, a total sample to minimum sample ratio of ~449 was used in a previous study according to the MIL-STD-105E table [14]. According to this ratio, a total of 170 was presumed as an appropriate sample size. At least 20 samples were collected systematically from each university to cover the different levels of study and various majors.

### 2.2. Questionnaire Design

A structured questionnaire was prepared to survey the general marine environmental knowledge (GMEK) of university students in Hong Kong (see Appendix A, parts A, B, C, D, E, and F). The questionnaire was divided into six parts: (A) General marine environmental knowledge, (B) marine environmental attitude, (C) land–sea interaction (LSI) conception, (D) methods of marine environmental education, (E) participation in marine-related courses and activities, and (F) demographic information.

Part A comprised 22 questions to assess the respondents’ current level of knowledge about the ocean and ocean environment. Two items (M1–M2) dealt with self-assessment of participants’ knowledge about the ocean and ocean environments. The next 20 questions (M3–M22) were intended to assess the participants’ general knowledge about the ocean and ocean environments. These 20 questions were formatted according to the seven principles of ocean literacy. Fourteen items were derived from the SOLE (Survey of Ocean Literacy and Experience) questionnaire [16], and the remaining six questions were developed to explore the students’ understanding of human–sea interaction more efficiently by highlighting local marine issues. These six newly developed questions also corresponded to the seven principles of ocean literacy and were validated by a pilot survey before final use. The distribution of items according to the seven ocean literacy principles is displayed in Table 2.

Part B was designed according to New Ecological Paradigm (NEP) suggested by Dunlap and Van Liere [39], which is an efficient tool for assessing pro-ecological world views and has been used successfully in many studies to date [14,40]. In this study, we adopted the NEP version used by Chen and Tasi [14], as they necessarily modified it for marine environments. A set of 10 modified NEP (A1–A10) statements was used in that study. Seven are positive statements, and the rest are negative statements. A 5-point Likert scale used to evaluate the respondent attitude statements; “strongly agree” was scored as 5, and “strongly disagree” was scored as 1 for positive statements; on the other hand, “strongly disagree” was scored as 5, and “strongly agree” was scored as 1 for negative statements.

Part C was designed to elicit students’ understanding of land–sea interactions and their opinions on including general marine environmental study (GMES) as a course curriculum at the university level. Three statements were added in the third part; statement P1 aimed to identify the respondents’ opinions about the inclusion of general marine environmental study as course curriculum, statement P2 explored respondents’ opinions about marine environmental knowledge for sustainable environmental management, and statement P3 was designed to identify respondents’ knowledge about the relationship between marine and terrestrial ecosystems. A five-point Likert scale was used to score participants’ responses.

Part D of the questionnaire dealt with the source of marine environmental education. Question E1 focused on the respondents’ major sources of marine environmental knowledge, and question E2 aimed to assess the respondents’ preference for the type of organization to achieve marine environmental knowledge. Questions E3 and E4 determined respondents’ interest in participating in certain pro-environmental activities and impediments to joining pro-environmental activities, respectively. This section was created and modified according to a study by Cheung et al. [41].

Part E of the questionnaire was used to determine respondent participation in marine-related courses and activities. Here, two options (Yes/No) were provided to select from based on the respondents’ level of engagement, with three questions in the section, namely D1, D2, and D3.

Finally, the last part (F) was about the demographic information of respondents: institution of study, gender, year of study, field of study and parents’ highest education level.

### 2.3. The Survey

Data were obtained through in-person and online surveys. The in-person survey was conducted using a no-interview format; a hard or soft copy (via QR code) of the questionnaire was provided to interested students in situ at the university canteen. In the online survey, the same questionnaire was emailed to interested students, and responses were recorded automatically via Google Drive. Face-to-face data collection was carried out from 16 May to 24 May 2017, and the online survey was kept open for one month from 1 May to 31 May 2017. A total of 205 students from eight universities completed the questionnaire successfully. The participation of at least 20 students was confirmed for every university. A total of 220 students were approached from different universities by the interviewer, and 198 students responded, demonstrating a high response rate of 90%. Ten out of 198 responses were excluded due to incomplete answers. Demographic information such as major of study, gender, year of study, and parents’ educational background was recorded. These demographic data are an essential factor for data analysis and simulation of the marine environmental consciousness of Hong Kong university students.

## 3. Statistical Analysis

All data analyses were performed using PASW 18 statistical software and Microsoft Excel 2013. Cronbach’s alpha (Cα) was applied to verify the reliability of questionnaire part B (Cα = 0.67) and part C (Cα = 0.74). A one-way ANOVA post hoc LSD test and t-test were applied to observe the significant mean differences between quantitative variables. Spearman’s rank correlation coefficient was used for data association and revealed a significant correlation among different quantitative variables. The Kolmogorov–Smirnov test was performed on the obtained dataset to observe the distribution pattern. Results showed a normal distribution of GMEK (*p* > 0.150), attitude (*p* > 0.150), and LSI (*p* > 0.150) data.

## 4. Result

### 4.1. Self-Evaluation

Students were asked for a self-evaluation of their knowledge about the marine environment, and the majority (M1: 59.04%; M2:62.76%) of students noted that they had “only a little” knowledge about ocean science (Table 3). A small number of students (M1: 3.72%; M2: 3.19%) claimed that they knew “A lot” about the marine environment (Table 3). A significant positive correlation (Spearman r = 0.901, p < 0.05) was observed between self-evaluated and actual knowledge for question M1. However, a significant negative correlation (Spearman r = 0.813, *p* < 0.05) was observed for question M2.

### 4.2. General Marine Environmental Knowledge

**Figure 1:**
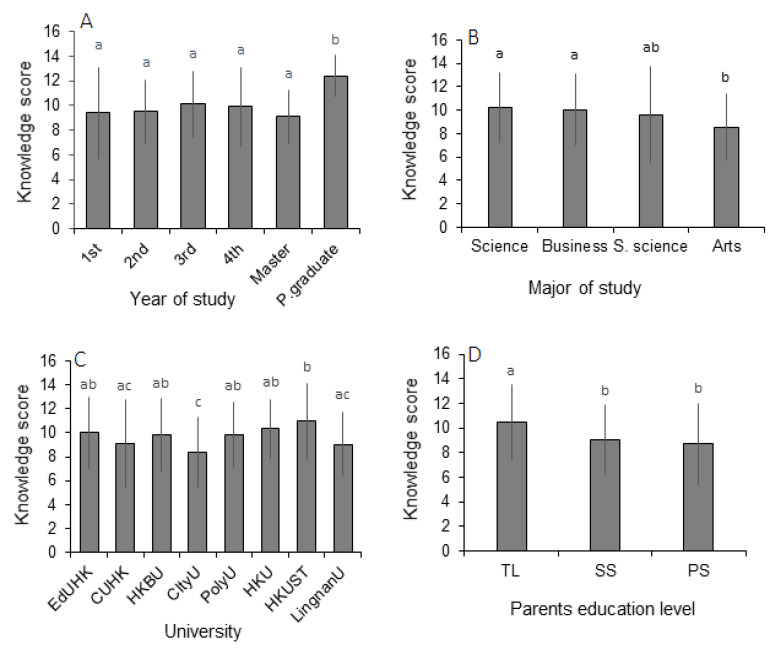
General marine environmental knowledge score (Mean ± SD) of students depending on different demographic variables: (**A**) year of study; (**B**) major of study; (**C**) major institution of study; (**D**) parents’ education level. Bars with different letters are significantly different (one-way post hoc LSD test; 95% level of confidence). TL = tertiary level of study, SS = secondary level of study, PS = primary level of study.

Respondents’ mean score on the multiple-choice questions was 9.69 out of 20 points. About half of the students scored 9.5, while only 27.12% of respondents scored the highest mean of 16.33 points, and 22.87% of respondents scored lowest average of 5 points. Students’ knowledge score was found to be quite promising for general ocean-science-related topics, including the unexplored area of ocean, the ecosystem, water cycle, biodiversity and sunlight–depth relationships. However, they had minimal knowledge as assessed by issue-based questions on topics such as ocean–carbon relationships; primary oil pollution sources; plastic pollution; ocean–technology relationships; and one of the most important pressing issues, i.e., the “White Chinese Dolphin”. Question M13 was designed for students to identify the current stresses on Chinese white dolphins, which is a popular local species with significant conservation concern, and only 23.9% of students answered it correctly (Table 4). Mean GMEK scores significantly varied depending on the level of study; postgraduate students achieved the highest scores, whereas students with a science background achieved the second-highest scores. In descending, educational backgrounds ranked as follows: science> business> social science> arts (Figure 1B). The group of students whose parents achieved a tertiary level of education scored significantly higher than the other two groups of students (parents’ education up to secondary and primary level; *p* < 0.01 and *p* < 0.05, respectively) (Figure 1D). Significant differences were also observed in GMEK scores among the students from different institutions (*p* < 0.01; *p* < 0.05; Figure 1C), and a non-significant difference was observed between genders (Table 5).

### 4.3. Environmental Attitudes

**Figure 2:**
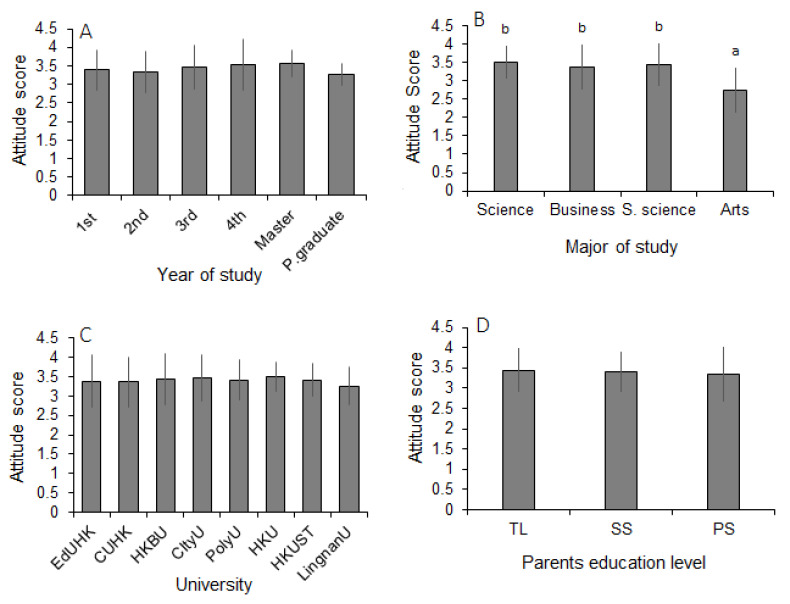
Attitude scores (mean ± SD) of students against different demographic variables: (**A**) year of study; (**B**) major of study; (**C**) major institution of study; (**D**) parents’ education level. Bars with different letters are significantly different (one-way post hoc LSD test; 95% level of confidence). TL = tertiary level of study, SS = secondary level of study, PS = primary level of study.

Ten NEP items were used in this study to measure students’ environmental attitudes, with a lowest score of 2.71 and a highest score of 4.07 (Table 6). The overall mean attitude score was calculated to be 3.4 ± 0.56 (Mean ± SD). However, students achieved moderately high attitude scores in ‘intergenerational equality (4.07)’, ‘abusing marine environment (4.03)’, ‘exploitation of marine resources (3.93)’, ‘harmony with the marine environment (3.80), and ‘human interference with the ocean (3.70)’. In contrast, they attained notably low scores on ‘balance of marine nature (2.6)’, ‘human right to modify the marine environment (2.84)’, and ‘usage of plants and animals (2.71)’ (Table 6). Mean attitude scores varied non-significantly against different demographic variables, such as year of study, educational institute (university), and parents’ education level (Figure 2A,C,D). However, these scores varied significantly depending on the field of study. Students majoring in art obtained significantly lower attitude scores than those with science, social science, and business backgrounds (Figure 2B). No significant variation was observed between male and female students’ mean attitude scores (Table 5).

### 4.4. Land–Sea Interaction and the Inclusion of Ocean Literacy Courses in Standard Curricula

Students’ understanding of LSI and interest in including marine study courses within standard curricula averaged approximately 3.5 (Table 7). Significant differences were also observed across two (major of study and university) out of four demographic variables. Students majoring in science earned a significantly higher score than those with arts (*p* < 0.05) and social science (*p* < 0.05) backgrounds (Table 7). However, the mean LSI scores varied non-significantly with respect to parents’ educational backgrounds and students’ level of study. The participants’ mean LSI scores according to level of study ranked in the following (descending) order: as master > postgraduate > fourth year > third year > first year > second year (Table 7). Overall, mean LSI score was significantly correlated with mean GMEK (*p* < 0.05) and attitude (*p* < 0.05) scores, albeit a non-significant weak positive relationship (r = 0.124) between the mean GMEK and attitude scores (Table 5).

## 5. Participation in Marine-Related Courses and Activities

In this section, we designed three questions to determine the students’ involvement in marine-protection-related courses, marine recreational activities, and marine conservation initiatives. The result showed that a small percentage (10.63%) of students had taken marine-protection-related courses (question no. D1; see Appendix A, part E), and about 90% of students mentioned that they had not taken any marine-environment-related courses. Regarding participation in any marine-related recreational activities (question no. D2; see Appendix A, part E), 55.31% of the students answered that they usually participate, and the remaining 44.68% of respondents did not have any experience in marine recreational activities. Students who participated in this study were asked about their participation in marine conservation initiatives (question no. D3; see Appendix A, part E). In reply, 30.85% of them mentioned that they had experience in marine conservation initiatives, and 69.14% answered that they did not participate in any conservative marine initiatives. Interestingly, the students who had experience in marine-related coursework and participated in marine conservation initiatives and recreational activities had higher attitude scores than those who did not participate in such activities (Table 8).

## 6. Methods of Marine Environment Education

According to the preferred methods of marine environmental education, the highest percentage of students (54.7%) mentioned web resources as a source of marine environmental knowledge, 35.10% alluded to their university, 17.20% to seminars, and 12.23% mentioned workshops (Table 9). A small number of students stated that they learned something about the marine environment at the primary and secondary levels. Some students also attributed their marine environmental knowledge to satellite channels (Discovery, Animal Planet, and National Geographic channels), different TV programs, documentaries, and encyclopedias. Students were asked about their preferred organization through which to take part in pro-environmental activities. In reply, 59.05% of respondents chose their university, 44.66% indicated non-governmental organizations (NGOs), chose 19.14% government, and 12.76% preferred their community organization (Table 9). The largest proportion of students who took part in this study believed that contributing to the environment (56.38%) and enhancing their knowledge (55.62%) are the key reasons for participating in pro-environmental activities. However, about 24.46% of students confirmed that they only participate in pro-environmental activities to increase their networks (24.46%) (Table 9). Participants mentioned an unsuitable schedule (49.46%), a lack of advertising for pro-environmental activities (41.48%), and expenses associated with participation (25.53%) as significant barriers to joining pro-environmental activities (Table 9).

## 7. Discussion

### 7.1. Ocean Literacy and Attitudes

This study shows that university students in Hong Kong possess moderate general marine environmental knowledge. Notably, students moderately understood issue-based current critical topics such as oil pollution sources, plastic pollution, and ocean–carbon relationships. However, they were less concerned or aware about pressing local issues such as habitat loss risks for white Chinese dolphins, although they displayed good knowledge of general ocean-science-related topics. Similar moderate ocean literacy was also observed in other localities, e.g., ocean literacy of Geek pre-service schoolteachers [19] and marine environmental awareness among Taiwanese university students [14]. In addition, students’ self-reported knowledge shows a mixed result in terms of specific questions, with a significant positive correlation between self-evaluated and actual knowledge for question M1 synchronized with their claims. However, a significant negative correlation for question M2 indicates that they have learned less about general marine environmental issues during primary and secondary school than they believe. Cheung et al. [41] found a similar disparity between self-reported and actual knowledge while studying residents’ environmental knowledge in Hong Kong. Therefore, it is essential to strengthen general marine environmental education at the primary and secondary levels.

The mean attitude score indicates a pro-environment attitude towards well disseminated environmental terms such as maintaining intergenerational equality, abuse of the marine environment, and harmony with nature. Still, students’ attitudes towards application-based items such as human rights to modify the marine environment and maintain the balance of marine nature are not pro-environmental. Previous research works, e.g., [14,42,43], reported similar results in terms of participants’ attitudes towards critical environmental issues. A weak positive correlation between attitude and GMEK indicates that knowledge does not always reflect attitudes [44]. In contrast, a strong positive relationship between attitude and LSI suggests that students with more positive attitudes can make further advanced decisions regarding land–sea interactions. In this study, the LSI section was designed to assess student responses with respect to further decision making such as inclusion of ocean literacy as a course curriculum, the urgency of marine sustainable management, and reducing land-based marine pollution sources. The students with more positive attitude scores were more likely to be successful in making these decisions. Therefore, it is crucial to concentrate on disseminating marine environmental knowledge in a practical way so that students will be concerned about essential marine environmental issues and involve themselves in pro-environmental practice.

### 7.2. Demographic Variables in Students’ Ocean Literacy

A significant difference was observed in students’ knowledge and attitudes across demographic variables (e.g., major of study, year of study, parents’ education, and gender). Participants from science backgrounds and studying at the graduate level scored more points than students from other backgrounds and those pursuing undergraduate degrees. However, senior students’ attitude scores were comparatively low, despite securing top scores in the general knowledge section. Other research findings also highlight the association of higher environmental knowledge with a higher education background [41,45,46]. Hence, knowledge is essential for developing some cognitive domains to act as pro-environmentalist but not always [41]. Fietkau and Kessel [47] demonstrated, in their model, that knowledge does not directly influence behavior but acts as a modifier of attitudes and values. On the other hand, the possibilities to act pro-environmentally, perceived consequences of behavior, and incentives for pro-environmental behavior are influencing factors for environmentally protective behaviors. According to another model, Blake [48] proposed that pro-environmental behavior and attitudes develop based on an individual’s responsibility, environmental concerns, internal individuality, and the situational influence of practicality. Our study results show a similar disparity between knowledge and attitude scores in terms of the level of study.

Parental education level was found to strongly affect students’ knowledge and attitudes in this study. Students whose parents had attained education up to the tertiary level were more knowledgeable than those whose had attained education up to the secondary and primary levels, with more pro-environmental attitudes and greater understanding of land–sea interactions. Khan [49] found a significant positive relationship between students’ academic achievements and parental education level. Campbell et al. [50] reported a higher average score among students with higher parental education levels. Yilmaz et al. [51] observed that undergraduate students’ high scores and behavior scores are correlated with parental education levels.

The mean GMEK and attitude scores varied non-significantly between male and female students. Male students secured slightly higher scores in the general knowledge section than females. They also obtained lower attitude scores and a less realistic understanding of land–sea interactions than female students. O’Brian [45] found a similar trend in her study conducted at Iowa State University. Mancl et al. [52] also observed differences in environmental knowledge level with respect to participants’ gender and ethnicity; they found a low level of ecological knowledge among female respondents. Tikka et al. [53] observed that female students were more likely to be more responsive to the environment, but male students scored higher in environmental knowledge. The results of the present study are in agreement with the results of these previous studies. The difference between females and males in attitudes are likely indicative of individual norms, which might explain their low attitude score or high general knowledge score [54,55]. However, the effects of different cultural and psychological contexts are still unclear, and further research is needed to comprehend the factors underlying this scenario.

### 7.3. University Variations in Students’ Ocean Literacy

Students with a science background achieved higher knowledge and attitude scores than students with other educational backgrounds. In particular, the students at institutions with broader-scale marine research facilities are more likely to attain higher ocean literacy scores. Marine-related activities such as hosting international seminars, symposia, and monthly newsletters may have helped students at such institutions to accumulate more knowledge about the marine environment. In contrast, the low scores listed for other institutions might be caused by the subject background effect and less access to extracurricular marine-related activities. It is usually assumed that students majoring in science would be more knowledgeable in marine environmental issues than those from other backgrounds, such as arts, business, and social sciences. Previous studies reported similar subject background effects on students’ general science knowledge at the university level [45,56]. Thus, more emphasis needs to be put on improving the marine literacy of non-science-background students during the dissemination of general marine education at the tertiary level.

### 7.4. Participating in Marine-Related Activities

Students’ participation in marine conservation and recreational activities and taking marine-protection-related courses were associated with positive attitudes towards the marine environment. Chen and Tasi [14] reported similar results in their study conducted at a Taiwanese university. Chiu et al. [57] observed that tourists’ eco-travel experiences can affect their environmentally responsible behavior. Lee and Moscardo [58] found that participation in natural activities and involvement in environmental practices led to decisive environmental actions. Kollmuss and Agyeman [43] stated that direct experiences are more influential on people’s behavior than indirect influences. To enhance students’ participation in marine-related activities, major marine science institutes and laboratories at different universities can take effective initiatives such as short-term educational programs, teacher training, and public outreach activities. Besides, other educational institutions can work collaboratively with universities to disseminate general marine environmental knowledge among students and local citizens.

The inclusion of GMES as a course curriculum might be the best-structured channel to enhance students’ ocean literacy. At present, most of the universities in Hong Kong have common core programs, which is promising for the development of students’ knowledge beyond their mainstream study. However, proper calibration is required to add GMES successfully in the tertiary education system as a course curriculum. The current research shows mixed responses from students regarding the inclusion of GMES as a course curriculum; 35.1% agreed, whereas 42.02% were unsure. More extensive studies are required with respect to this aspect from the primary to university level to determine students’ current level of ocean literacy. School-level assessments (primary and secondary) of ocean literacy have been increasingly conducted across the globe in the last few decades (e.g., the United States, United Kingdom, Mexico, Canada, South Africa, Greece, Korea, Taiwan, and China) [15,21,24,59,60,61,62,63,64,65]. In contrast, only a few studies have been conducted to assess students’ ocean literacy at the university level [14,19]. Therefore, more studies are required at the university level to better identify challenges and effective ways to include GMES as a course curriculum. Proper integration between the primary school and university levels is needed to ensure a sustainable approach of including GMES as a course curriculum.

A tiny portion (10.63%) of the total participants mentioned that they had taken marine-related courses. Similarly, Chen and Tasi [14] observed that a high percentage of students do not take marine-science-related courses, and they urged an increase in enrollment in marine-related courses at the tertiary level. Strang et al. [66] mentioned that one cannot be considered “science literate” without being “ocean literate.” Undoubtedly, becoming a science-literate citizen is time-demanding, and ocean literacy is a crucial topic to understand the total environmental system, alongside terrestrial environmental knowledge and their interactions. Besides taking marine-related courses, students were also asked to choose their secondary source of ocean literacy. They choose web resources, their university, seminars, and workshops as the best channels for environmental education. Therefore, a structured way of enriching university web platforms, as well as arranging monthly seminars and workshops, might effectively disseminate ocean literacy. In the question on barriers, students highlighted an unsuitable schedule, expense, lack of promotion, and interest as significant obstacles to participating in marine pro-environmental programs. The authorities responsible for arranging seminars, symposia, or other related environmental awareness promotion activities should ensure student-friendly access and scheduling. Students’ interest is vital to ensure their voluntary participation in awareness programs; therefore, marine environmental knowledge should be taught in a structured way to elevate their interest. Respondents also mentioned their university, NGOs, and government institutes as leading organizations through which to participate in pro-environmental activities. Hence, universities can play a crucial role by including relevant course curricula, enriching web resources, and arranging outreach activities to teach ocean literacy to their students. NGOs and government institutions can also play a key role and work collaboratively with universities to disseminate general marine environmental knowledge through a well-structured pathway.

## 8. Conclusions

The current study shows moderate marine environmental knowledge and attitudes among Hong Kong university students, with some weaknesses in understanding the land–sea interaction, especially issue-based current critical topics. However, this study identified universities as the most preferred institutes and web sources as the most preferred platform for acquiring marine environmental education. Therefore, developing a web-based education platform through a collaborative effort among higher education institutes may help students from different backgrounds to easily acquire general marine environmental knowledge. A small portion of students mentioned that they had experience in taking marine-related courses. Still, their marine environmental knowledge level was moderate, and a large percentage of students did not have any experience in marine-related activities. Students from different study backgrounds generally have limited experience in taking marine-related classes. However, the inclusion of general marine science as a subsidiary course at the university level might be a time-demanding way of fostering ocean literacy, although further research is required on this perspective to evaluate the efficacy of adding general marine environmental study as a course curriculum at the tertiary level.

## Figures and Tables

**Table 1:** Sampling location and sample size.

University	Area	Participants
The Education University of Hong Kong (EduHK)	Tai Po, New Territories	24
The Chinese University of Hong Kong (CUHK)	Sha Tin, New Territories	32
Hong Kong Baptist University (HKBU)	Kowloon Tong, Kowloon	20
City University of Hong Kong (CityU)	Kowloon Tong, Kowloon	21
Polytechnic University of Hong Kong (Poly U)	Hong Hum, Kowloon	22
The University of Hong Kong (HKU)	Pok Fu Lam, Hong Kong Island	23
Hong Kong University of Science and Technology (HKUST)	Clear Water Bay, New Territories	25
Lingnan University	Tuen Mun, New Territories	21
Total: 8 Universities		188

**Table 2:** Group of questions associated with the seven principles of ocean literacy.

Ocean Literacy Principle	Number of Questions	Question Range
1. The Earth has one big ocean with many features	3	M3–M5
2. The ocean and life in the ocean shape the features of earth	3	M6–M8
3. The ocean has a major influence on weather and climate	2	M9–M10
4. The ocean made the Earth habitable	1	M11
5. The ocean supports a great diversity of life and ecosystems	3	M12–M14
6. The ocean and humans are inextricably interconnected	6	M15–M20
7. The ocean is largely unexplored	2	M21–M22
Total	20	

**Table 3:** Respondents’ self-evaluation of marine environmental knowledge.

	M1. In General, to What Extent Do You Think You Know About General Marine Environmental Knowledge?	M2. To What Extent Do You Think You Learned about the Marine Environment in Your School Life (Primary and Secondary)?	Actual Knowledge Score M1 (Mean ± SD)	Actual Knowledge Score M2 (Mean ± SD)
A lot (%)	3.72	3.19	10.71 ± 4.46	11.16 ± 5.06
A fair amount (%)	20.21	15.42	10.02 ± 2.76	9.51 ± 2.89
Only a little (%)	59.04	62.76	9.74 ± 3.10	9.56 ± 2.99
Practically nothing (%)	17.02	18.61	8.74 ± 2.73	10.02 ± 3.04

**Table 4:** Students’ responses to general marine environmental knowledge questions.

Question	Correct (%)	Incorrect (%)
**M3.** Which one is the world’s largest ecosystem on the earth?	71.3	21.7
**M4.** The ocean contains the earth’s_____	37.8	62.2
**M5.** Approximately how much of the earth’s water is fresh and unfrozen (neither ice nor ocean)?	38.9	69.1
**M6.** What are the major pollutants found on sea beach?	65.4	34.6
**M7.** Water moves from the ocean to the atmosphere to the land and back again to the ocean by a process called	79.3	20.7
**M8.** Sea level changes over time have_____	47.3	52.7
**M9.** Which are the primary sources of oil in the ocean?	17.6	82.4
**M10.** The ocean controls weather and climate by dominating which following earth’s systems?	51.6	48.4
**M11.** What happens to sunlight in the ocean as depth increases?	72.3	27.7
**M12.** Where is a greater diversity of living organisms found?	73.9	26.1
**M13.** Which of the following is not the main threat for Chinese white dolphin?	23.9	76.1
**M14.** What produces most of the earth’s oxygen and is also a potential source of agar and bioethanol?	46.3	53.7
**M15.** The ocean dominates the earth’s carbon cycle. Approximately how much of all the carbon dioxide in the atmosphere is absorbed by the ocean?	17.1	83.9
**M16.** What is the main source of marine pollution?	40.4	59.6
**M17.** Which of the following Marine reptiles is in the significant threat of fishing bycatch, illegal trade, and habitat loss?	54.3	45.7
**M18.** Which form of plastic can be very dangerous when it transports along with the marine food web?	31.3	68.6
**M19.** Which following technology tools do ocean scientists rely on more to explore the ocean?	36.2	63.8
**M20.** Which of the following metal pollution in seawater could be very much harmful to human health via consumption of fish?	71.8	28.2
**M21.** Which of the following ocean ecosystems provides habitat for one-third of all marine species?	42.1	57.9
**M22.** The ocean is the last and largest unexplored place on earth. How much of the ocean remains unexplored?	59.0	41.0

**Table 5:** Mean knowledge and attitude scores based on the students’ gender. t *p*-values are at a 95% confidence level. Correlations among general marine environmental knowledge, attitude, and land–sea interaction scores. t-v indicates t-value.

Topic	Gender	N	Mean	SD	Df	t-v	*p*-Value
Knowledge score	Male	106	9.96	3.18	186	1.35	*p* > 0.05
	Female	82	9.3	2.22			
Attitude score	Male	106	3.35	0.56	186	−1.48	*p* > 0.05
	Female	82	3.48	0.56			
**Correlation among GMEK, Attitude, and LSI**							
				GMEK	Attitude	LSI	
			GMEK	1.000			
			Attitude	0.124	1.000		
			LSI	0.179 *	0.384 **	1.000	

* Correlation is significant at the 0.05 level. ** Correlation is significant at the 0.01 level. GMEK = general marine environmental knowledge; LSI = land–sea interaction.

**Table 6:** Mean attitude scores.

Statements	Mean	SD
**Positive statements**		
**A1.** The present generation should ensure that the environment is maintained or enhanced for the benefit of future generations	4.07	0.99
**A2.** The earth is like a spaceship with only limited room and resources	3.62	1.30
**A3.** To maintain a healthy and sustained marine ecosystem, we will have to control utilization of marine living resources	3.93	1.14
**A4.** When humans interfere with the marine environment, it often produces disastrous consequences	3.70	1.11
**A5.** Humans must live in harmony with marine nature in order to survive	3.8	1.04
**A6.** The balance of marine nature is very delicate and easily upset	2.6	1.21
**A7.** Mankind is severely abusing the marine environment	4.03	0.93
**Negative statement items**		
**A8.** Humans have the right to modify the marine environment to suit their needs	2.84	1.20
**A9.** Humans need not adapt to the marine environment because they can remake it to suit their needs	2.77	1.28
**A10.** Plants and animals exist primarily to be used by humans	2.71	1.30
**Mean attitude score**	3.42	0.56

**Table 7:** Mean scores of three statements related to land–sea interaction (LSI) and inclusion of marine study courses.

Statement			Mean	SD
**P1.** “General Marine environmental study should be included as a course curriculum at the university level especially for the students who aren’t majoring in marine science”			3.18	1.02
**P2.** “Marine environmental knowledge is highly important for sustainable environmental management”			3.52	1.12
**P3.** “Land-based activities highly influence the marine environment; therefore, understanding the marine environment is very important to fill up the knowledge gap between land-based pollution and Marine pollution” **LSI score against different demographic variables**			3.57	1.04
Demographic variable		N		
Year of study	1st	55	3.39	0.85
	2nd	57	3.26	0.86
	3rd	31	3.39	0.76
	4th	26	3.63	0.97
	Master	11	3.81	0.74
	P. graduate	8	3.65	0.96
Major of study	Science	96	3.62 ^a^	0.82
	Arts	54	3.17 ^b^	0.81
	Business	27	3.42 ^ab^	0.92
	S. science	10	2.96 ^b^	0.85
University	EdUHK	24	3.60 ^ac^	0.85
	CUHK	32	3.26 ^bcd^	0.97
	HKBU	20	3.64 ^ad^	0.86
	CityU	21	3.34 ^ab^	0.82
	PolyU	22	3.11 ^bc^	0.88
	HKU	23	3.77 ^a^	0.81
	HKUST	25	3.59 ^ac^	0.67
	LingnanU	21	3.06 ^b^	0.77
Parents education	TL	90	3.42	0.91
	SS	77	3.48	0.80
	PS	21	3.21	0.81

Mean values with different letters are significantly different (one-way post hoc LSD test) at a 95% confidence level.

**Table 8:** Students’ attitude scores according to their marine-related course experience, marine recreational activities, and conservation activities. Appendix A, part E provides details about questions D1, D2, and D3. Independent sample *t*-test represents significant differences among students’ attitude scores regarding their responses for questions D1, D2, and D3. t-v indicates t-value.

Question	Response	%	Attitude Score				
			Mean	SD	Df	t-v	*p*-Value
D1	YES	10.63	3.5	0.4	187	3.3	*p* < 0.01
	NO	89.36	3.3	0.5			
D2	YES	55.31	3.5	0.5	187	3.3	*p* < 0.01
	NO	44.68	3.3	0.5			
D3	YES	30.85	3.5	0.5	187	3.3	*p* < 0.01
	NO	69.14	3.3	0.5			

**Table 9:** Major organizations, aims, and barriers to joining pro-environmental activities.

Choice	Percentage (%)
Preferred organizations through which to participate in pro-environmental activities	
University	59.04
NGO	44.66
Government	19.14
Community organization	12.76
Principal aims of joining a certain pro-environmental activities	
Enhancing knowledge	55.32
Increasing personal network	24.46
Contribution to the environment	56.30
For career development	7.97
Peer pressure	8.64
It is a university activity	10.10
Incentives	5.31
Major barriers to joining pro-environmental activities	
Too expensive	25.53
Bad weather conditions	13.8
Not interesting enough	35.63
The schedule is not suitable	49.46
Lack of promotion	41.48

## Data Availability

Data are available on request form the corresponding author. Due to privacy issue data are not made publicly available.

## Appendix A. Survey Questionnaire

**Part A**

**M1. In general, to what extent do you think you know about general marine environmental knowledge?**

(a) A lot (b) A fair amount (c) Only a little (d) practically nothing

**M2. To what extent do you think you learned about the marine environment in your school life?**

(a) A lot (b) A fair amount (c) Only a little (d) practically nothing

**M3. Which one is the world’s largest ecosystem on the earth?**

(a) Terrestrial ecosystem (b) Desert ecosystem (c) Marine Ecosystem (d) Forest ecosystem (e) both a & b

**M4. The ocean contains the earth’s**

(a) Flattest plains (b) Tallest Mountain (c) Deepest valley (d) all are in the ocean (e) none are in the ocean

**M5. Approximately how much of the earth’s water is fresh and unfrozen (neither ice nor ocean)?**

(a) >50% (b) 40–50% (c) 20–30% (d) 10–20% (e) 3% (f) 1%

**M6. What are the major pollutants found on sea beach?**

(a) Crude oil (b) Plastic waste (bottle, packet) and cans (c) Heavy metal (d) None of them (e) Both a & b.

**M7. Water moves from the ocean to the atmosphere to the land and back again to the ocean by a process called**

(a) Water shed (b) Hurricane (c) Water cycle (d) Tsunami (e) Cyclone (f) Perfect storm

**M8. Sea level changes over time have**

(a) Increased and decreased continental shelves

(b) Created and destroyed inland seas

(c) Shaped the surface of land

(d) All of these

(e) None of these

**M9. Which are the primary sources of oil in the ocean?**

(a) Used motor oils washed into storm drains

(b) Leaks from refineries and pipelines

(c) Evaporation from oil cargoes, which return to the ocean by rain out

(d) Leaks from offshore oil rigs

(e) None of these sources put oil in the ocean

**M10. The ocean controls weather and climate by dominating which following earth’s systems?**

(a) Energy (b) Plants (c) Water (d) Carbon (e) Answer a, c, & d (f) None of these systems

**M11.** What happens to sunlight in the ocean as depth increases?

(a) Increases with depth (b) Decreases with depth (c) Stays the same (d) Increase & decrease (e) None of these

**M12. Where is a greater diversity of living organisms found?**

(a) On the land (b) In the ocean (c) Both equally

**M13. Which of the followings is not the main threat for Chinese white dolphin?**

(a) Habitat loss (b) Pollution (c) Turbidity of water (d) Intense marine traffic (e) None of them

**M14. What produces most of the earth’s oxygen and is also a potential source of agar and bioethanol?**

(a) Forests (b) Plants (algae) in the ocean (c) Both equally (d) None of them

**M15. The ocean dominates the earth’s carbon cycle. Approximately how much of all the carbon dioxide in the atmosphere is absorbed by the ocean?**

(a) 30% (b) 50% (c) 60% (d) 70% (e) 90% (f) 97%

**M16. What is the main source of marine pollution?**

(a) Anthropogenic/Land base (b) Natural/Geological (c) Cosmic source (d) None of them (e) Both a & b

**M17. Which of the following Marine reptiles is in the significant threat of fishing bycatch, illegal trade, and habitat loss** **?**

(a) Marine Iguana (b) Sea turtle (c) Saltwater crocodile (d) Sea snake

**M18. Which form of plastic can be very dangerous when it transports along with the marine food web?**

(a) Nano/micro plastic (b) Big plastic particle (c) Both of them (d) None of them

**M19. Which following technology tools do ocean scientists rely on more to explore the ocean?**

(a) Buoys (b) Satellites (c) Subsea observatories (d) Unmanned submersibles (e) All of these (f) Both c and d

**M20. Which of the following metal pollution in seawater could be very much harmful to human health via consumption of fish** **?**

(a) Copper (Cu) (b) Zinc (Zn) (c) Iron (Fe) (d) Mercury (Hg) (e) None of them

**M21. Which of the following ocean ecosystems provides habitat for one-third of all marine species?**

(a) Coral reef (b) Seagrass meadow (c) Mangrove Forest (d) Open ocean e) Estuary

**M22. The ocean is the last and largest unexplored place on earth. How much of the ocean remains unexplored?**

(a) 30% (b) 50% (c) greater than 90% (d) less than 5% (e) 65%

**Part B**

**For every statement, please indicate whether you strongly agree (SA), mildly agree (MA), are unsure (U), mildly disagree (MD) or strongly disagree (SD) with it. There is no correct or wrong answer. (Give the tick (√ or X) mark on the chosen answer)**

**Statement (Attitude)**	**SA**	**MA**	**U**	**MD**	**SD**
**Positive statement items**					
**A1.** The present generation should ensure that the environment is maintained or enhanced for the benefit of future generations					
**A2.** The earth is like a spaceship with only limited room and resources					
**A3.** To maintain a healthy and sustained marine ecosystem, we will have to control utilization of marine living resources					
**A4.** When humans interfere with the marine environment, it often produces disastrous consequences					
**A5.** Humans must live in harmony with marine nature in order to survive					
**A6.** The balance of marine nature is very delicate and easily upset					
**A7.** Mankind is severely abusing the marine environment					
**Negative statement items**					
**A8.** Humans have the right to modify the marine environment to suit their needs					
**A9.** Humans need not adapt to the marine environment because they can remake it to suit their needs					
**A10.** Plants and animals exist primarily to be used by humans					

**Part C**

**Give the tick (√ or X) mark on chosen answer**

**P1. “General Marine environmental study should be included as a course curriculum at the university level especially for the students who aren’t majoring in marine science”**

(a) Strongly agree (b) Mildly agree (c) Unsure (d) Disagree (e) Strongly disagree

**P2. “Marine environmental knowledge is highly important for sustainable environmental management”**

(a) Strongly agree (b) Mildly agree (c) Unsure (d) Disagree (e) Strongly disagree

**P3. “Land-based activities highly influence the marine environment; therefore, understanding the marine environment is very important to fill up the knowledge gap between land-based pollution and Marine pollution”**

(a) Strongly agree (b) Mildly agree (c) Unsure (d) Disagree (e) Strongly disagree

**Part D**

**Respondent Marine environment education related questions. Give the tick (√ or X) mark on chosen answer**

**E1. What are the major sources of your marine environmental education** **(can choose multiple options)?**

1. University	2. Seminar
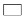
3. Exhibition	4. Visits
5. Workshop	6. Web resources
7. Eco/Geo	8. None 9. Other (Specify)……………………..

**E2. You would usually join pro-environmental activities organized by which of the following bodies (can choose multiple options)?**

1. Your University	2. Government
3. NGO	4. Your community
5. Other (Please specify):…………………	

**E3. What is your principle aim to join a certain pro-environmental activity (can choose multiple options)?**

1. Enhancing Knowledge	2. Increasing personal network
3. Contribution to the environment	4. It is a university activity.
5. For career development	6. Incentives
7. Peer pressure	8. Other (Specify)………….

**E4. Which barrier prohibits you from joining a pro-environmental activity (can choose multiple options)**?

1. Too expensive	2. The schedule is not suitable
3. Bad weather conditions	4. High demand on physical ability
5. Not interesting enough	6. lack of promotion
7. Others (specify): ______	

**Part E**

**D1. Have you ever taken any marine protection-related courses?**

o Yes o No

**D2. Have you ever participated in any marine recreational activities? (e.g., surfing, boating, snorkeling, diving)**

o Yes o No

**D3. Have you ever participated in any marine conservation initiative? (e.g., beach cleanup, marine ecotourism)**

o Yes o No

**Part F**

**Demographic information**

**What is your gender?**

o Male

o Female

**Which university are you studying in?**

o The University of Hong Kong

o The Chinese University of Hong Kong

o Hong Kong University of Science and Technology

o The Hong Kong Polytechnic University

o City University of Hong Kong

o Hong Kong Baptist University

o Education University of Hong Kong

o Lingnan University

o Other (specify):______

**Years of your study**

o 1 o 2 o 3 o 4 o Other (specify): _______

**What is your parent’s highest educational level?**

o Primary school o Secondary school o Tertiary level or above
